# Effects of Habitual Caffeine Intake, Physical Activity Levels, and Sedentary Behavior on the Inflammatory Status in a Healthy Population

**DOI:** 10.3390/nu12082325

**Published:** 2020-08-03

**Authors:** Lluis Rodas, Aina Riera-Sampol, Antoni Aguilo, Sonia Martínez, Pedro Tauler

**Affiliations:** 1 Research Group on Evidence, Lifestyles and Health, University of the Balearic Islands, 07122 Palma, Spain; lluisrodas@hotmail.com; 2 Research Group on Evidence, Lifestyles and Health, Department of Nursing and Physiotherapy, University of the Balearic Islands, 07122 Palma, Spain; ana.riera@uib.es; 3 Research Group on Evidence, Lifestyles and Health, Department of Nursing and Physiotherapy, Health Research Institute of the Balearic Islands (IdISBa), University of the Balearic Islands, 07122 Palma, Spain; aaguilo@uib.es; 4 Research Group on Evidence, Lifestyles and Health, Department of Fundamental Biology and Health Sciences, Health Research Institute of the Balearic Islands (IdISBa), University of the Balearic Islands, 07122 Palma, Spain

**Keywords:** caffeine, coffee, physical activity, siting time, inflammation, body fat

## Abstract

Low-grade chronic inflammation is associated with many chronic diseases and pathological conditions. The aim of the present study was to determine the effect of regular caffeine intake, physical activity levels, and sedentary behavior on the inflammatory status in healthy participants. In total, 112 men and 132 women aged 18 to 55 years and belonging to the staff and student population of the University of the Balearic Islands volunteered to participate in this descriptive cross-sectional study. Plasma concentrations of pro-inflammatory and anti-inflammatory markers were measured. Weight, height, and body composition (bioelectrical impedance) were determined. Caffeine intake, physical activity levels and sitting time, and diet quality were determined using questionnaires. Statistical regression analysis showed that caffeine intake was a negative predictor of C-reactive protein (CRP) (*p* = 0.001). Body fat percentage was positively associated with CRP (*p* < 0.001) and inversely associated with adiponectin (*p* = 0.032) and interleukin (IL)-10 levels (*p* = 0.001). Visceral fat was the main predictor for IL-6 (*p* < 0.001) and tumor necrosis factor (TNF)-α (*p* < 0.001). Sitting time was found to be the main, inverse, predictor for IL-10 (*p* < 0.001), and a positive predictor for TNF-α (*p* < 0.001). In conclusion, regular caffeine consumption induced very limited anti-inflammatory effects. Sedentary behavior and body fat accumulation induced significant pro-inflammatory effects.

## 1. Introduction

Low-grade chronic inflammation is characterized by chronically (two to three-fold) increased concentrations of several cytokines such as interleukin (IL)-6 and tumor necrosis factor (TNF)-α, as well as other pro-inflammatory substances such as C-reactive protein (CRP) [1]. Fat accumulation has been suggested to be the main reason for increased levels of these pro-inflammatory markers [2,3]. Many chronic diseases such as arthritis, type-2 diabetes or cancer, and pathological conditions such as insulin resistance or atherosclerosis have been found to be associated with low-grade systemic inflammation [2,3,4].

Physical inactivity has also been linked to low-grade systemic inflammation and subsequent increased risk for the development of chronic diseases [5]. On the other hand, regular exercise has been associated with an anti-inflammatory status, characterized by higher levels of anti-inflammatory markers such as IL-10 and adiponectin, and lower levels of pro-inflammatory cytokines, including IL-6, TNF-α, and IL-1β [2,4]. It has been pointed out that exercise-induced anti-inflammatory effects could be produced by reduced fat accumulation [2]. Interestingly, sedentary behavior, commonly defined as any sitting activity with a low energy expenditure, has been also linked to low-grade inflammation, independently of physical activity levels or adiposity [6,7,8,9].

Caffeine, due to its widespread presence in foods such as coffee, tea, and chocolate, and its stimulant effects, is highly consumed around the world. In vitro studies have suggested an anti-inflammatory role for caffeine, mainly inhibiting TNF-α production [10]. Regarding in vivo studies, only a few have addressed the effects of caffeine supplementation on blood inflammatory markers in humans [11]. These studies used a single dose of caffeine or used coffee as supplement when longer interventions were tested, reporting slight anti-inflammatory effects from the supplementation [12,13]. However, when coffee is used, many more components than caffeine alone are included in the supplement, as coffee is rich in bioactive compounds with antioxidant and anti-inflammatory properties, mainly chlorogenic acids [14,15]. In a similar way, when effects of habitual caffeine intake are analyzed it is impossible not to consider coffee consumption and the contribution of other coffee components, because in most countries, including Spain, coffee has been reported to be the main dietary source of caffeine [16]. In this regard, it has been shown that regular coffee intake is associated with reduced risk of type 2 diabetes [17,18] and metabolic syndrome [19] among other clinical conditions where low-grade inflammation and oxidative stress is involved in their development [11]. Furthermore, studies have shown anti-inflammatory effects of regular coffee consumption [20,21,22,23].

To our knowledge the effect of habitual caffeine intake on the inflammatory status of people performing different levels of physical activity, while taking into consideration the amount of sitting time, in a healthy young population has not yet been determined. Therefore, the aim of the present study was to determine the effect of regular caffeine intake, physical activity levels and sedentary behavior on the inflammatory status in healthy participants. Because physical activity and also caffeine, or coffee, have been suggested to induce anti-inflammatory effects, it could be expected that physically active participants consuming caffeine could present a more anti-inflammatory profile.

## 2. Materials and Methods

### 2.1. Study Design and Participants

Two hundred and forty-four participants (112 men and 132 women) volunteered to participate in this descriptive cross-sectional study. Participants were recruited among healthy students, workers, researchers and lecturers from the University of the Balearic Islands. All the participants were informed of the purpose and demands of the study before giving their written consent to participate. The protocol was in accordance with the Declaration of Helsinki for research of human participants and was approved by the Balearic Islands Clinical Investigation Ethics Committee (IB 2399/14 PI).

Participants were enrolled after fulfilling all inclusion criteria and presenting none of the exclusion criteria. Participants could be included if they were currently healthy, aged between 18 and 55 years and to have maintained constant physical activity levels (regardless of how high or low the level of activity) and sedentary behavior within the previous two months. Exclusion criteria were: smokers, professional, elite and those athletes with a habitual participation in endurance and ultra-endurance events, significant body weight fluctuations within the previous two months (±2 kg), regular alcohol or drugs consumption, and habitual consumption, or consumption within the 2 weeks preceding the study, of anti-inflammatory medication. Two hundred and forty-eight participants were recruited, but blood samples could not be obtained from four, leading to the final number of participants indicated above.

### 2.2. Laboratory Visit

Participants arrived at the laboratory between 08:00 and 10:00 h following an overnight fast of approximately 12 h. They had been previously informed about the study demands and about the inclusion and exclusion criteria. They had also been instructed to abstain from any moderate-vigorous intensity exercise during the 24 h before coming to the laboratory. Information about the study was given again to the participants, they completed an inclusion/exclusion criteria questionnaire and they then signed an informed consent form. Each participant was asked to empty their bladder before body mass, height and body composition were recorded. Participants then sat quietly for 10 min and completed questionnaires to determine physical activity levels, caffeine intake and adherence to the Mediterranean diet, before a blood sample was taken. The women were also asked about the date of their last menstruation. Seated venous blood samples were collected in suitable vacutainers with ethylenediaminetetraacetic acid (EDTA). Plasma was obtained from the blood samples within 30 min after blood collection by centrifugation (15 min, 1000× *g*, 4 °C). Plasma aliquots were stored at −70 °C until measurements were performed.

### 2.3. Anthropometrical Measurements

Height was measured to the nearest 0.5 cm using a stadiometer (Seca 220 (CM) Telescopic Height Rod for Column Scales, Seca gmbh, Hamburg). Body weight, percentage of body fat mass, and rating of visceral fat were measured using a Body Composition Analyzer (Bioelectrical Impedance Analysis, TANITA MC-780MA, TANITA Europe BV, Amsterdam, The Netherlands). Body weight was measured to the nearest 0.1 kg. The visceral fat measurement using this methodology provides a rating from 1 to 59, with values from 1 to 12 considered healthy (arbitrary units and information provided by the manufacturer, https://tanita.eu/help-guides/products-manuals/). Body mass index (BMI) was calculated as weight (kg) divided by height (m) squared (kg·m^−2^).

### 2.4. Questionnaires

Physical activity levels were determined using the standard short form of the validated International Physical Activity Questionnaire (IPAQ), thus providing quantitative information on physical activity levels in metabolic equivalents (MET)-h·week^−1^. Total weekly physical activity time and daily sitting time were also determined.

Diet quality was measured as the Adherence to the Mediterranean diet using a simplified assessment of adherence to the Mediterranean Diet (14-item questionnaire), previously developed and validated for the Spanish population [24]. Each item was scored as 0 or 1. A global score of 9 or higher indicates a good adherence to the Mediterranean Diet.

Habitual caffeine intake was measured using a self-reported questionnaire previously developed and used by our group [25,26]. The frequency intake of common products containing caffeine was ascertained, and caffeine daily consumption determined by using the caffeine content of each product [16]. Products included in the questionnaire were coffee preparations, including decaffeinated coffee, instant coffee (as cups or spoons), tea, chocolate, cola drinks, energy or stimulant beverages, and pharmaceutical caffeine supplements. Coffee intake was estimated from the intake of each coffee preparation contained in the questionnaire. The most common preparations in Spain were considered: espresso, “cortado” (espresso coffee, one serving, with a shot of milk), and “café con leche” (white coffee, or espresso coffee, one serving, with the remaining half of a cup filled with milk or steamed milk). Because a dose-response plasma appearance of the chlorogenic and phenolic acids contained in coffee has been shown after instant coffee ingestion [27], and because of its caffeine content, instant coffee was also considered as a coffee preparation. When applicable, the number of instant coffee servings was determined considering the number of spoons indicated by participants and that each serving contained 3.4 g of instant coffee [27]. Therefore, the total coffee intake, in number of servings, was determined considering all preparations indicated above.

### 2.5. Measurement of Inflammatory Markers

Plasma IL-10, IL-6, IL-1β, TNF-α, adiponectin, and CRP concentrations were determined to describe the inflammatory status of participants. These inflammatory markers were measured using commercially available solid-phase sandwich enzyme-linked immunosorbent assays and performed according to the manufacturers’ instructions. Concentrations of IL-10, IL-6, and TNF-α were measured using Invitrogen high sensitivity kits (ThermoFisher SCIENTIFIC, Waltham, MA, USA). IL-10 was measured using the “IL-10 Human ELISA Kit, High Sensitivity” (BMS215HS); IL-6 was measured using the “IL-6 Human ELISA Kit, High Sensitivity” (BMS213HS); and TNF-α was measured using the “TNF alpha Human ELISA Kit, High Sensitivity” (BMS223HS). IL-1β, adiponectin and CRP were measured using RayBio^®^ kits (RayBiotech, Norcross, GA, USA). IL-1β was measured using the “Human IL-1 beta ELISA” (ELH-IL1b); Adiponectin was measured using the “Human Adiponectin (Acrp30) ELISA” (ELH-Adiponectin); and CRP was measured using the “Human CRP ELISA” (ELH-CRP). All kits were exclusive for human samples (plasma, serum, and culture supernatants), and micro-plates were supplied with wells pre-coated with the specific antibody. For all assays, the absorbance was measured spectrophotometrically on a microplate reader (PowerWavei; BioTek, Winooski, VT, USA), and the concentration of each cytokine was calculated by comparison with a calculation curve established in the same measurement.

### 2.6. Statistical Analysis

Statistical analysis was carried out using IBM SPSS Statistics 22.0 software (SPSS/IBM, Chicago, IL, USA). All the data were tested for their normal distribution (Kolmogorov–Smirnov test). The results are expressed as means and standard deviations (SD), or median and interquartile ranges as specified. Percentages were also used when required. Student’s t-test for unpaired data or the Mann-Whitney U test were used to evaluate differences between sexes. Kruskal-Wallis one-way ANOVA was used to determine the effect of the menstrual cycle on cytokine, adiponectin and CRP concentrations in women. Because no effect of menstrual cycle was observed (results not shown), this variable was not included in the following main analysis. The existence of significant bivariate correlations between the main variables was ascertained by determining Pearson correlation coefficients. These correlations were determined for the whole sample and for male and female participants separately. Multiple linear regression analysis, using the stepwise procedure, was applied to determine the relation between each dependent variable (logarithmic transformed IL-10, IL-6, IL-1β, TNF-α, adiponectin, and CRP concentrations) and independent and control variables. Habitual caffeine intake, IPAQ score and sitting time were included in the analysis as independent variables. Sex, body fat percentage, visceral fat rating, and diet quality, measured as the Adherence to the Mediterranean diet, were included in the analysis as control variables. Kruskal-Wallis one-way ANOVA was used to analyze IL-10 and TNF-α values depending on sitting time categorized in tertiles. A post-hoc power analysis calculation was performed (G*Power 3.1.9.4. Universität Kiel, Germany) for the regression analysis (*n* = 244, eight predictors, α = 0.05). The power statistical calculation reported values higher than 90% for all significant regression reported. Statistical significance was accepted at *p* < 0.05.

## 3. Results

### 3.1. General Characteristics of Participants in the Study

Table 1 shows the general characteristics of participants in the study stratified per sex. The study population was, on average, young, with a BMI value within the normal weight range, a Mediterranean diet score slightly under the threshold for a good adherence, and with a higher number of women. Regarding anthropometrical characteristics, 20.5% of participants were overweight (BMI between 25 and 30 kg·m^−2^), and 3.7% were obese (BMI higher than 30 kg·m^−2^). On the other hand, 2.2% of participants showed an unhealthy visceral fat rating. Regarding caffeine intake, 4.9% of participants did not take caffeine, and 17% of participants took more than 400 mg per day (which is considered the higher-end value for a throughout the day, not in a single dose, safe consumption in healthy people [16]). Regarding coffee intake, 22.1% of participants reported no coffee intake, and 7% of participants reported an intake greater than 3 servings. Men reported non-significantly higher physical activity levels, both when expressed in METs and in hours. No significant differences between sexes were observed in the sitting time.

### 3.2. Sources of Caffeine Consumption

Among caffeine consumers, coffee was the main caffeine source for participants in the study (Table 2). No significant differences were found between male and female participants.

### 3.3. Concentration of Inflammatory Markers

Table 3 shows cytokine concentrations of all participants and categorized by sex. Significant differences were observed for IL-6, TNF-α, CRP, and adiponectin, with higher values in men for IL-6 (*p* = 0.003) and TNF-α (*p* = 0.005), and higher values in women for CRP (*p* < 0.001) and adiponectin (*p* < 0.001).

### 3.4. Bivariate Correlations between Dependent and Independent Variables

Table 4 shows bivariate correlations between all dependent and independent (continuous) variables. Positive correlations were found for IL-6 (*p* = 0.001) and TNF-α (*p* = 0.014) with age. However, IL-10 was inversely correlated with age (*p* = 0.032). Caffeine intake was inversely correlated with CRP levels (*p* = 0.044). Physical activity levels were correlated with IL-10 (*p* = 0.002). Sitting time showed a positive correlation with TNF-α (*p* < 0.001) and an inverse correlation with IL-10 levels (*p* < 0.001). Percentage of body fat was correlated with CRP (*p* < 0.001), while it was inversely correlated with IL-10 (*p* = 0.001) and adiponectin (*p* < 0.001). An inverse correlation was found between visceral fat rating and IL-10 (*p* = 0.013). However, visceral fat showed positive correlations with IL-6 (*p* < 0.001) and TNF-α (*p* < 0.001). IL-1β levels were correlated with the adherence to the Mediterranean diet (*p* = 0.039).

Table 5 shows bivariate correlations between all dependent and independent variables in female participants. An inverse correlation was found between IL-10 and age (*p* = 0.020). However, a positive correlation was found for IL-6 with age (*p* = 0.023). Physical activity levels were correlated with IL-10 (*p* = 0.033). Sitting time showed a positive correlation with TNF-α (*p* = 0.001) and an inverse correlation with IL-10 levels (*p* = 0.001). Percentage of body fat showed a positive correlation with CRP (*p* < 0.001). Positive correlations were found for IL-6 (*p* = 0.037) and TNF-α (*p* = 0.049) with visceral fat. No correlation was found for adherence to the Mediterranean diet or caffeine intake with any of the dependent variables.

Table 6 shows bivariate correlation between all dependent and independent variables in male participants. A positive correlation was found between TNF-α and age (*p* = 0.042). Physical activity levels were positively correlated with IL-10 (*p* = 0.010) and inversely correlated with TNF-α (*p* = 0.005). Sitting time showed a positive correlation with TNF-α (*p* = 0.047) and an inverse correlation with IL-10 levels (*p* < 0.001). Percentage of body fat was correlated with IL-6 (*p* = 0.001), TNF-α (*p* < 0.001), and CRP (*p* < 0.001), while it was inversely correlated with adiponectin (*p* < 0.001) and IL-10 (*p* = 0.002). Visceral fat was correlated with IL-6 (*p* = 0.002), TNF-α (*p* < 0.001), and CRP (*p* < 0.001), and it was inversely correlated with IL-10 (*p* = 0.008). No correlation was found for adherence to the Mediterranean diet or caffeine intake with any of the dependent variables.

### 3.5. Multivariable Linear Regression Analysis

Table 7 shows the results of the regression analysis for IL-10. Regression analysis revealed that sitting time (*p* < 0.001) and the percentage of fat (*p* = 0.001) were negative predictors for IL-10 levels, while physical activity was a positive predictor (*p* = 0.028). Sitting time was found to be the main predictor (change in R^2^ 0.106 vs. 0.040 for fat mass and 0.017 for physical activity).

Table 8 shows de-regression analysis results for adiponectin. Sex was revealed as the main predictor for adiponectin (change in R^2^ 0.097, *p* < 0.001), with higher values for females, as has been indicated above. Percentage of fat mass was inversely associated with adiponectin (change in R^2^ 0.017, *p* = 0.032).

Table 9 shows the results of the regression analysis for IL-6. Visceral fat was revealed as the only significant predictor for IL-6 (*p* < 0.001), with a positive association.

Sitting time (change in R^2^ 0.044, *p* < 0.001,) and visceral fat rating (change in R^2^ 0.112, *p* < 0.001) were found as positive predictors for TNF-α concentrations, with visceral fat as the main predictor (Table 10).

Table 11 shows the results of the regression analysis for CRP. Percentage of fat mass was found as the main predictor for CRP (change in R^2^ 0.182, *p* < 0.001), showing a positive association. Caffeine intake was shown to be a negative predictor for CRP (change in R^2^ 0.037, *p* = 0.001). The regression analysis did not report any significant predictor for IL-1β.

### 3.6. Effects of Sitting Time on IL-10 and Tumor Necrosis Factor (TNF) -a Concentrations

**Figure 1:**
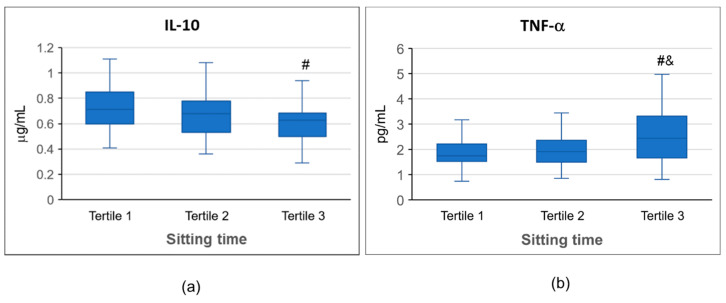
IL-10 (**a**) and TNF-α (**b**) plasma concentrations categorized by sitting time tertiles. Median, 25th, 75th percentile, and lowest and highest values are shown for IL-10 and TNF-α values. # indicates significant differences to first tertile. & indicates significant differences to second tertile. IL: interleukin; TNF: tumor necrosis factor.

Figure 1 shows values of IL-10 (a) and TNF-α (b) categorized by sitting time tertiles. A significant effect of sitting time was found for both IL-10 (*p* = 0.001) and TNF-α (*p* < 0.001). IL-10 values in the third tertile (longer sitting time, 9 to 15 h) were significantly lower (*p* = 0.001) than in the first tertile (shortest sitting time, 1 to 5 h). TNF-α values in the third tertile were significantly higher than in the second tertile (*p* = 0.004) and in the first tertile (*p* < 0.001).

## 4. Discussion

The main finding of the present study was that in a healthy population, low caffeine intake could exert a slight anti-inflammatory effect characterized by lower CRP plasma levels. Body fat, both total and visceral, and sedentary behavior have been shown to be important and independent inflammatory predictors, inducing higher levels of pro-inflammatory markers, but also decreased levels of anti-inflammatory markers.

Participants in the study population were, on average, young and with a higher number of women. These characteristics reflect properly the university population, with higher figures for young female students. Caffeine intake observed in the present study was similar to previous studies in Spain, and similar, or slightly lower to the average value, when participants of a similar age from most occidental countries were considered [16,28,29,30]. Concerning this consumption, coffee remains the main source for caffeine intake, a common finding in most European countries, except for the UK and Ireland [16]. As coffee preparations in Spain are quite different from the ones in other countries, it becomes difficult to compare coffee intake observed in the present study with the habitual intake of other countries. For these reasons, caffeine intake, rather than coffee intake, has been used as the main variable for the analysis, and also as an indicator of coffee consumption.

Previous studies, both observational and clinical trials, have reported that coffee intake increases adiponectin levels [13,20]. The mechanism involved seems to be the stimulatory effect of caffeine on the expression of the peroxisome proliferator activated receptor γ (PPARγ), which positively elevates the adiponectin concentration [31]. In fact, a previous study reported increased levels of adiponectin after a supplementation with caffeinated coffee but no effect with decaffeinated coffee [13]. However, a higher daily intake of coffee (four cups, or more than 600 mg of caffeine) than that observed in the present study, seems to be required to increase adiponectin levels [20]. Therefore, and as suggested, low amounts of coffee or caffeine consumption may be the reason for not observing a significant effect of caffeine intake on adiponectin [32] but also on IL-10, TNF-α, and IL-6 concentrations in the present study. It should also be considered that some studies reporting positive effects of regular coffee consumption on adiponectin levels were performed in overweight participants [13], while in the present study only ~24% of participants were overweight or obese; or in older populations [20]. Therefore, it is possible that the protective effect of coffee was emphasized in such conditions.

The association between CRP, which is considered an appropriate marker for low grade systemic inflammation [33], and coffee intake has been recently reviewed [34]. Despite this review suggesting that on average, coffee consumption is not associated with CRP levels, some studies have reported a protective healthy effect because decreased levels of CRP have been observed in participants ingesting at least a daily cup of coffee [20,21,22,23]. Results of the present study are in agreement with this observation as it was observed that caffeine consumption is inversely associated with CRP levels. Therefore, it seems that in contrast with the other inflammatory markers analyzed, low regular coffee intake could be enough to prevent higher CRP levels. It is worth noting however, that decaffeinated coffee seems to also be effective in decreasing CRP levels, therefore other components of coffee rather than caffeine itself, could contribute to this effect [22]. It has been suggested that chlorogenic acid, the main phenolic acid in coffee with antioxidant and anti-inflammatory properties [14], can play an important role in the reduction of inflammatory factors such as CRP [35]. The low, on average, BMI of participants in the present study could have been an important factor that influenced the inverse association between coffee intake and CRP, as all studies reporting the protective anti-inflammatory effect of coffee consumption were performed in populations with low BMIs [20,21,22,23]. In fact, it has been suggested that the different BMI of populations considered in previous studies could be a key factor for the inconsistent results found regarding this association [34]. Interestingly, in the present study, and in agreement with previous reports [36,37], a positive association was found between body fat mass and CRP. Therefore, our data indicates that, in the context of an average low BMI, caffeine consumption, probably as a coffee consumption marker, presents an inverse association with CRP, inducing the opposite effect of body fat mass accumulation.

Inverse associations were found between percentage of body fat and anti-inflammatory markers such as adiponectin and IL-10. Adiponectin is an anti-inflammatory adipokine secreted almost exclusively from adipose tissue [38], and low adiponectin levels have been associated with body fat accumulation and obesity, leading to increased risk of inflammation [3]. It is striking that in the present study this potential negative effect was observed in young and healthy participants, with an incidence of obesity as low as 3.7%. In addition, and despite the fact women present a relatively greater percentage body fat, adiponectin levels were found to be higher in women, which is in agreement with previous results, mainly when lean populations have been considered [39].

The anti-inflammatory IL-10 has not been commonly measured when associations between fat mass and inflammation have been investigated. In the present study, and in the same line of results indicated above, IL-10 was inversely associated with body fat mass. It has been reported that fat accumulation is accompanied by adipose tissue infiltration by pro-inflammatory immune cells (T2), increased release of pro-inflammatory markers such as TNF-α, decreased production of anti-inflammatory markers, such as IL-10, and the development of the low-grade systemic inflammatory state described above [3]. Within this picture, and regarding adipose tissue, visceral fat has been suggested to play an important role [2]. In the present study positive associations were found between visceral fat and TNF-α and also between visceral fat and IL-6. Previous studies, using different approaches to measure visceral fat, have shown similar associations [40,41,42]. Whether TNF-α and IL-6 levels are more dependent on total body fat or visceral fat remains to be elucidated, because some studies have reported associations with both parameters [37,42,43]. However, taken together, the opposite associations found for IL-10 and, on the other hand, TNF-α and IL-6 with regard to fat content, total or visceral, could reflect the predominant anti-inflammatory production when fat content is low as well as the predominant pro-inflammatory release when fat is accumulated.

In the present study only a small effect of physical activity levels on the IL-10 concentrations were observed. However, more strong associations were found for sedentary time: a positive association with TNF-α and an inverse association with IL-10, which, together, define a pro-inflammatory picture induced by sedentary behavior and independent of physical activity levels. It is possible that the average moderate levels reported could limit the effects of physical activity. However, previous studies showed that sedentary behavior was linked to low-grade inflammation, independently of physical activity levels or adiposity [6,7,8,9]. This observation is in agreement with results obtained in the present study and suggests an independent link between sitting time and low-grade inflammation [7]. To our knowledge, the association between sedentary time and IL-10 has not been reported, as previous studies focused on the pro-inflammatory markers. This novel result could indicate that sedentary behavior, independent of fat mass and physical activity, influences levels of both pro-inflammatory and anti-inflammatory compounds.

This study presents some limitations that should be acknowledged. In addition to the limitations due to the observational nature, the current study was limited to cross-sectional data from a single university population. However, the patterns of physical activity and sedentary behavior observed in the present study could be applied to other populations, even outside the university. Both sedentary time and physical activity levels, two of the main variables of the study, were self-reported. However, the IPAQ, which is a widely used and validated questionnaire, was utilized to collect these data, as has been done in previous studies using the same or similar questionnaires [6,9]. Regarding sedentary time, an important limitation was that the number, duration, and frequency of sedentary breaks were not recorded. In this regard, it has been reported that these breaks could modify to some extent associations between sedentary time and inflammatory markers [8], at least when sitting time is not too long [7]. Furthermore, methodology used to determine both percentage of fat mass and visceral fat rating has been reported to present some limitations [44]. Finally, despite the statistical power analysis revealed high power for most of the associations found, these associations could be considered weak. However, there is a concordance between most of them, highlighting the pro-inflammatory role of sedentary behavior and fat mass accumulation.

## 5. Conclusions

The limited effects of caffeine or coffee intake observed in the present study could be explained by relatively low caffeine and coffee intakes. However, this relatively low caffeine or coffee consumption could slightly prevent CRP increases induced by increased fat mass. Sedentary behavior and body fat accumulation, even within the young, healthy and, on average, normal weight participants in the present study induced pro-inflammatory effects. However, only slight effects of physical activity levels were observed. Interestingly, both sedentary behavior and fat accumulation induced lower levels of the essential anti-inflammatory cytokine IL-10. Future studies should be performed to determine coffee intake needed to observe greater health effects and, also, to determine the mechanism linking sedentary behavior to inflammation.

## Figures and Tables

**Table 1:** General characteristics of participants in the study.

	All (*n* = 244)	Men (*n* = 112)	Women (*n* = 132)	*p* Value
Age (years)	32.1 ± 10.8	33.4 ± 10.8	31.1 ± 10.8	0.098
Weight (kg)	66.9 ± 13.3	76.3 ± 11.4	58.9 ± 8.7	**<0.001** *
Height (cm)	170.0 ± 9.0	177.0 ± 6.5	164.0 ± 6.0	**<0.001** *
BMI (kg·m^−2^)	23.0 ± 3.4	24.3 ± 3.3	22.0 ± 3.2	**<0.001** *
Fat mass (%)	23.3 ± 7.9	18.3 ± 6.3	27.6 ± 6.5	**<0.001** *
Visceral fat rating	4.01 ± 3.30	5.66 ± 3.68	2.63 ± 2.12	**<0.001** *
Mediterranean diet score	8.18 ± 1.91	8.22 ± 1.93	8.15 ± 1.91	0.771
Caffeine intake (mg·day^−1^)	164.3 ± 143.4	174.7 ± 152.4	155.5 ± 135.3	0.298
Caffeine intake (mg·kg body weight^−1^·day^−1^)	2.48 ± 2.18	2.33 ± 2.18	2.60 ± 2.18	0.346
Coffee intake (servings·day^−1^)	1.32 ± 1.35	1.41 ± 1.47	1.24 ± 1.25	0.311
Physical activity levels (METs-hour·week^−1^)	43.8 ± 36.1	48.7 ± 33.7	39.7 ± 37.5	0.054
Physical activity (hours·week^−1^)	9.18 ± 8.50	9.90 ± 7.36	8.57 ± 9.35	0.224
Sitting time (hours·day^−1^)	6.96 ± 2.77	7.20 ± 3.09	6.77 ± 2.46	0.235

Values are expressed as means ± standard deviations. * indicates significant differences between sexes (with *p* significant values also in bold) (*p* < 0.05), Student’s t-test for unpaired data. BMI: body mass index.

**Table 2:** Sources of caffeine intake among those participants consuming caffeine.

Source	All (*n* = 228)	Men (*n* = 105)	Women (*n* = 123)	*p* Value
Coffee (%)	67.5 ± 37.1	69.6 ± 37.4	66.0 ± 36.9	0.538
Tea (%)	10.1 ± 19.8	8.9 ± 19.4	11.0 ± 20.1	0.425
Cola drinks (%)	12.8 ± 24.7	14.0 ± 26.8	11.8 ± 22.9	0.506
Chocolate (%)	7.9 ± 20.4	5.2 ± 14.1	10.2 ± 24.2	0.054
Energetic drinks (%)	1.6 ± 7.6	2.2 ± 9.0	1.0 ± 6.1	0.241
Sport products (%)	0.1 ± 0.7	0.1 ± 1.0	n.d.	0.175

Values are expressed as means ± standard deviations and represent the caffeine contribution in percentage of each source with respect to total caffeine intake. No differences between men and women were found, Student’s t-test for unpaired data. n.d.: non detected (no consumption reported).

**Table 3:** Cytokine concentrations of participants in the study.

Inflammatory Marker	All (*n* = 244)	Men (*n* = 112)	Women (*n* = 132)	*p* Value
IL-10 (μg·mL^−1^)	0.65 (0.54, 0.77)	0.65 (0.55, 0.83)	0.66 (0.54, 0.74)	0.333
IL-6 (μg·mL^−1^)	2.17 (1.44, 3.04)	2.38 (1.84, 3.12)	1.89 (1.14, 2.99)	**0.003** *
IL-1β (pg·mL^−1^)	3.84 (2.66, 6.29)	4.02 (2.88, 6.40)	3.58 (2.43, 6.00)	0.205
TNF-α (pg·mL^−1^)	1.98 (1.52, 2.68)	2.11 (1.64, 3.04)	1.76 (1.49, 2.51)	**0.005** *
CRP (μg·mL^−1^)	3.50 (1.81, 5.67)	2.46 (1.58, 4.28)	4.26 (2.42, 6.34)	**<0.001** *
Adiponectin (μg·mL^−1^)	5.78 (3.62, 7.96)	4.59 (3.12, 6.22)	6.72 (4.77, 8,78)	**<0.001** *

Values are expressed as median (25th, 75th percentile). * indicates significant differences between men and women (with *p* values in bold) (*p* < 0.05), Mann Whitney U test. IL: interleukin; TNF: tumor necrosis factor; CRP: C-reactive protein.

**Table 4:** Bivariate correlations between dependent and independent variables (all participants).

	Age	%Fat	Vis Fat	MD	Caffeine	PA	Sit
IL-10	−0.138 (**0.032** *)	−0.219 (**0.001** *)	−0.159 (**0.013** *)	−0.083 (0.197)	−0.094 (0.142)	0.195 (**0.002** *)	−0.325 (<0.001*)
IL-6	0.205 (**0.001** *)	0.063 (0.329)	0.290 (**<0.001** *)	−0.032 (0.615)	0.089 (0.164)	−0.037 (0.561)	0.003 (0.957)
IL-1β	0.006 (0.932)	0.071 (0.267)	0.052 (0.417)	0.132 (**0.039** *)	−0.006 (0.930)	−0.009 (0.889)	−0.030 (0.639)
TNF-α	0.157 (**0.014** *)	0.086 (0.180)	0.335 (**<0.001** *)	−0.023 (0.717)	0.067 (0.299)	−0.081 (0.208)	0.249 (**<0.001** *)
CRP	0.009 (0.889)	0.427 (**<0.001** *)	0.115 (0.074)	−0.109 (0.088)	−0.129 (**0.044** *)	−0.037 (0.563)	−0.039 (0.549)
Adiponectin	−0.107 (0.095)	−0.222 (**<0.001** *)	−0.117 (0.072)	−0.012 (0.429)	−0.087 (0.182)	−0.041 (0.520)	−0.003 (0.963)

Pearson correlation coefficient and (*p* value) is shown (*n* = 244). * indicates significant correlations (with *p* values in bold) (*p* < 0.05). % Fat: percentage of total body fat; Vis fat: visceral fat rating; MD: adherence to the Mediterranean diet; Caffeine: caffeine intake; PA: physical activity levels. Sit: sitting daily time. Logarithmic transformations of dependent variables were used. IL: interleukin; CRP: C-reactive protein; TNF: tumor necrosis factor.

**Table 5:** Bivariate correlations between dependent and independent variables in females.

	Age	%Fat	Vis Fat	MD	Caffeine	PA	Sit
IL-10	−0.203 (**0.020** *)	−0.133 (0.128)	−0.170 (0.052)	−0.045 (0.605)	−0.098 (0.265)	0.185 (**0.033** *)	−0.281 (**0.001** *)
IL-6	0.198 (**0.023** *)	0.162 (0.064)	0.182 (**0.037** *)	−0.046 (0.597)	0.054 (0.539)	−0.100 (0.254)	0.017 (0.844)
IL-1β	−0.005 (0.959)	−0.011 (0.902)	0.048 (0.588)	0.157 (0.072)	−0.016 (0.854)	−0.028 (0.754)	−0.091 (0.300)
TNF-α	0.096 (0.274)	0.164 (0.060)	0.172 (**0.049** *)	−0.062 (0.479)	−0.034 (0.699)	−0.016 (0.856)	0.297 (**0.001** *)
CRP	−0.066 (0.455)	0.330 (**<0.001** *)	0.158 (0.070)	−0.097 (0.267)	−0.155 (0.077)	0.030 (0.729)	0.048 (0.582)
Adiponectin	−0.111 (0.243)	0.063 (0.510)	−0.053 (0.579)	0.152 (0.109)	−0.027 (0.773)	−0.106 (0.268)	−0.062 (0.513)

Pearson correlation coefficient and (*p* value) is shown (*n* = 132). * indicates significant correlations (with *p* values in bold) (*p* < 0.05). % Fat: percentage of total body fat; Vis fat: visceral fat rating; MD: adherence to the Mediterranean diet; Caffeine: caffeine intake; PA: physical activity levels. Sit: sitting daily time. Logarithmic transformations of dependent variables were used. IL: interleukin; CRP: C-reactive protein; TNF: tumor necrosis factor.

**Table 6:** Bivariate correlations between dependent and independent variables in males.

	Age	%Fat	Vis Fat	MD	Caffeine	PA	Sit
IL-10	−0.100 (0.296)	−0.295 (**0.002** *)	−0.249 (**0.008** *)	−0.120 (0.207)	−0.102 (0.286)	0.242 (**0.010** *)	−0.369 (**<0.001** *)
IL-6	0.180 (0.058)	0.300 (**0.001** *)	0.293 (**0.002** *)	−0.024 (0.803)	0.130 (0.164)	−0.023 (0.806)	−0.042 (0.662)
IL-1β	−0.002 (0.980)	−0.054 (0.577)	−0.011 (0.913)	0.094 (0.323)	−0.006 (0.953)	0.004 (0.953)	0.026 (0.784)
TNF-α	0.193 (**0.042** *)	0.353 (**<0.001** *)	0.378 (**<0.001** *)	0.115 (0.226)	0.148 (0.118)	−0.262 (**0.005** *)	0.188 (**0.047** *)
CRP	0.166 (0.079)	0.366 (**<0.001** *)	0.381 (**<0.001** *)	−0.122 (0.200)	−0.074 (0.440)	−0.051 (0.594)	0.076 (0.424)
Adiponectin	−0.107 (0.095)	−0.222 (**<0.001** *)	−0.117 (0.072)	−0.012 (0.429)	−0.087 (0.182)	−0.041 (0.520)	−0.003 (0.963)

Pearson correlation coefficient and (*p* value) is shown (*n* = 112). * indicates significant correlations (with *p* values in bold) (*p* < 0.05). % Fat: percentage of total body fat; Vis fat: visceral fat rating; MD: adherence to the Mediterranean diet; Caffeine: caffeine intake; PA: physical activity levels. Sit: sitting daily time. Logarithmic transformations of dependent variables were used. IL: interleukin; CRP: C-reactive protein; TNF: tumor necrosis factor.

**Table 7:** Multiple linear regression for IL-10.

Variable	B	95% CI	β	t	*p* Value
Age	−0.002	(−0.006, 0.002)	−0.104	−1.706	0.089
Sex	0.010	(−0.060, 0.080)	0.042	0568	0.570
Fat mass	−0.003	(−0.005, −0.001)	−0.189	−3.171	**0.002** *
Visceral fat rating	−0.002	(−0.014, 0.010)	−0.066	−1.346	0.180
Adherence to MD	−0.005	(−0.013, 0.003)	−0.078	−1.324	0.187
Caffeine intake	0.000	(−0.001, 0.000)	−0.080	−1.336	0.183
Physical activity	0.000	(0.000, 0.001)	0.136	2.218	**0.028** *
Sitting time	−0.012	(−0.017, −0.007)	−0.281	−4.607	**<0.001** *

Model: R: 0.405; R^2^ 0.164; Adjusted R^2^ 0.153; *p* < 0.001; * indicates significant predictors (with *p* values in bold) (*p* < 0.05). IL: interleukin; MD: Mediterranean diet; CI: confidence intervals.

**Table 8:** Multiple linear regression for adiponectin.

Variable	B	95% CI	β	t	*p* Value
Age	0.000	(−0.004, 0.004)	−0.019	−0.285	0.776
Sex	0.194	(0.124−0.265)	0.407	5.426	**<0.001** *
Fat mass	−0.005	(−0.009, −0.001)	−0.162	−2.156	**0.032** *
Visceral fat rating	0.004	(−0.004−0.012)	0.050	0.443	0.658
Adherence to MD	0.008	(−0.006, 0.022)	0.065	1.075	0.283
Caffeine intake	0.000	(−0.001, 0.000)	−0.068	−1.092	0.276
Physical activity	0.000	(−0.001, 0.000)	−0.012	0.010	0.846
Sitting time	0.003	(−0.007, 0.013)	0.039	0.637	0.585

Model: R^2^ 0.115; Adjusted R^2^ 0.107; *p* < 0.001; * indicates significant predictors (with *p* values in bold) (*p* < 0.05). MD: Mediterranean diet; CI: confidence intervals.

**Table 9:** Regression analysis for IL-6.

Variable	B	95% CI	β	t	*p* Value
Age	0.001	(−0.003, 0.005)	0.013	0.151	0.880
Sex	−0.035	(−0.075, 0.005)	−0.073	−1.055	0.292
Fat mass	−0.001	(−0.003, 0.001)	−0.028	−0.439	0.661
Visceral fat rating	0.021	(0.012, 0.030)	0.290	4.701	**<0.001** *
Adherence to MD	−0.005	(−0.017, 0.007)	−0.044	−0.706	0.481
Caffeine intake	0.000	(0.000, 0.001)	0.039	0.579	0.563
Physical activity	−0.001	(−0.002, 0.001)	−0.059	−0.948	0.344
Sitting time	−0.003	(−0.011, 0.005)	−0.033	−0.532	0.595

Model: R^2^ 0.084; Adjusted R^2^ 0.080; *p* < 0.001; * indicates significant predictors (with *p* values in bold) (*p* < 0.05). IL: interleukin; MD: Mediterranean diet; CI: confidence intervals.

**Table 10:** Multiple linear regression for tumor necrosis factor (TNF) -α.

Variable	B	95% CI	β	t	*p* Value
Age	−0.002	(−0.005, 0.001)	−0.108	−1.327	0.186
Sex	−0.014	(−0.128, 0.100)	−0.041	−0.608	0.544
Fat mass	−0.001	(−0.011, 0.010)	−0.023	−0.367	0.714
Visceral fat rating	0.016	(0.010, 0.022)	0.309	5.166	**<0.001** *
Adherence to MD	0.001	(−0.009, 0.012)	0.007	0.119	0.905
Caffeine intake	0.000	(−0.001, 0.000)	−0.060	−0.936	0.350
Physical activity	0.000	(−0.001, 0.000)	−0.025	−0.984	0.326
Sitting time	0.013	(0.004, 0.019)	0.211	3.538	**<0.001** *

Model: R^2^ 0.156; Adjusted R^2^ 0.149; *p* < 0.001; * indicates significant predictors (with *p* values in bold) (*p* < 0.05). TNF: tumor necrosis factor; MD: Mediterranean diet; CI: confidence intervals.

**Table 11:** Multiple linear regression for C-reactive protein (CRP).

Variable	B	95% CI	β	t	*p* Value
Age	−0.002	(−0.010, 0.006)	−0.039	−0.601	0.549
Sex	−0.016	(−0.082, 0.050)	−0.021	−0.288	0.774
Fat mass	0.018	(0.013, 0.022)	0.454	7.885	**<0.001** *
Visceral fat rating	0.006	(−0.022, 0.034)	0.063	0.980	0.328
Adherence to MD	−0.011	(−0.029, 0.007)	−0.070	−1.219	0.224
Caffeine intake	−0.002	(−0.003, −0.001)	−0.195	−3.379	**0.001** *
Physical activity	0.000	(−0.004, 0.004)	0.023	0.407	0.684
Sitting time	0.000	(−0.013, 0.013)	0.011	0.187	0.852

Model: R^2^ 0.219; Adjusted R^2^ 0.213; *p* < 0.001; * indicates significant predictors (with *p* values in bold) (*p* < 0.05). CRP: C-reactive protein; MD: Mediterranean diet; CI: confidence intervals.
